# Characterization of Phenolic Profile in Milk Obtained by Ewes Fed Grape Pomace: Reflection on Antioxidant and Anti-Inflammatory Status

**DOI:** 10.3390/biom13071026

**Published:** 2023-06-22

**Authors:** Francesca Bennato, Andrea Ianni, Eleonora Oliva, Nicola Franceschini, Lisa Grotta, Manuel Sergi, Giuseppe Martino

**Affiliations:** 1 Department of Biosciences and Technology for Food, Agriculture and Environment, University of Teramo, 64100 Teramo, Italy; 2 Department of Biotechnological and Applied Clinical Sciences, University of L’Aquila, Via Vetoio 1, 67100 L’Aquila, Italy; 3 Chemistry Department, University La Sapienza, 00185 Rome, Italy

**Keywords:** ewes’ milk, phenolic compounds, metalloproteases, molecular docking, milk antioxidant status, milk anti-inflammatory status

## Abstract

The aim of the present work was to evaluate if the use of grape pomace (GP) in the feeding of dairy ewes can improve the content of phenolic compounds (PCs) in the milk and affect the anti-inflammatory and antioxidative status of the milk. For this purpose, 46 ewes were randomly assigned to two groups of 23 animals each: a control group (Ctrl) that received a standard diet and an experimental group (GP+), whose diet was been formulated with 10% GP on a dry matter (DM) basis. At the end of the 60 days of the trial, from 10 ewes selected randomly from each group, individual milk samples were collected and analyzed for the identification and the quantification of phenolic compounds through an ultra-high-performance liquid chromatography system, and milk anti-inflammatory and antioxidative status were evaluated by enzyme-linked immunosorbent assay, determining the activity of GPx and CAT and the levels of IL-1 and TNFα. In addition, gelatinolytic activity of Type IV collagenases (MMP-2/MMP-9) was evaluated by the fluorometric method and zymographic approach. The results obtained showed that the diet with GP affects the phenolic profile of milk, inducing milk enrichment of phenolic compounds without, however, having a significant impact on milk antioxidant and inflammatory status. However, a lower activity of MMP-9 was found in GP+ milk. The use of the molecular docking approach showed the ability of luteolin to approach the catalytic pocket of the enzyme, interfering with the recruitment of the substrate, and therefore, slowing down their hydrolytic activity.

## 1. Introduction

In the last years, in the zootechnical field, particular attention has been given to the use of non-conventional byproducts rich in phenolic compounds (PCs), which could be interesting for the feeding and nutrition of small dairy ruminants as a source of bioactive compounds. The data reported in literature is often contrasting because of the effects on PCs intake on animal health and consequently on the quality of dairy products depend on their chemical characteristics, rumen digestion, and post-absorption. Due to PCs binding to dietary proteins, moderate levels of PCs in the diets of ruminants can reduce protein ruminal degradation, leading to an increase in amino acid flow to the small intestine [1,2,3]. The ability of PCs to modulate the rumen biohydrogenation of polyunsaturated fatty acids (PUFA) [4] leads to an improvement in the quality of the lipid fraction of dairy products [5] by increasing the concentration of beneficial fatty acids (e.g., PUFA, vaccenic, and rumenic acids) and increasing the oxidative stability of products [6,7].

Among byproducts rich in PCs, in the last years, there has been a growing interest in the use of grape pomace (GP) in small ruminant feeding. GP, the main solid byproduct of the wine industry, is characterized by a significant amount of substances that can be considered beneficial to health [8]. The most important constituents of GP are unsaturated fatty acids, fibers, minerals, vitamins, and PCs that mainly (about 70%) remain in pomace after the winemaking process [9,10]. As reported by Makris et al. [11], GP is characterized by high concentrations of extractable polyphenols (approximately 10% on a dry matter (DM) basis), specifically phenolic acids, anthocyanins, catechins, procyanidins, flavonols, and stilbenes; however, the exact composition is strongly dependent on grape variety and also tends to vary in different parts of the grape [10]. Generally, the red varieties are characterized by high concentrations of anthocyanins, while in white varieties the flavan-3-ols (gallocatechin, procyanidin B1, procyanidin B2, procyanidin B4, procyanidin C1, catechin, and epigallocatechin) have been reported to be the most abundant polyphenols [12,13].

Most of the PCs isolated from GP have exhibited interesting biological properties in both in vitro and in vivo studies. In humans, the ability of PCs to act as an antioxidant [14] and an anti-inflammatory has been evidenced [15]. The positive effects of polyphenols on animals’ health have been evidenced by improvements in the cell-mediated immune response [16], reduction in inflammatory processes [17], and improvements in the antioxidant status of animals [18,19].

The mechanisms of the antioxidant activity of PCs are based on their structure and include radical scavenging ability, electron donation, or chelation of metal ions in vitro [20]. Moreover, such direct radical scavenging activity or reducing power of PCs is observed only at concentrations significantly higher than the physiological levels found in vivo. Increasing evidence shows that the in vivo antioxidant and anti-inflammatory effects of PCs and their metabolites arise from their ability in modulating cellular signalling transductions. Even low concentrations of dietary PCs have been shown to be able to restore the redox homeostasis and prevent systemic or localized inflammation by enhancing activities of the antioxidant enzymes superoxide dismutase (SOD), catalase (CAT), and glutathione peroxidase (GPx), whose expression is modulated by a key transcription factor nuclear factor erythroid-related factor (Nrf2). Nrf2 can be activated by ROS at the cellular level, translocate into nucleus, and regulate antioxidant-responsive elements (ARE), mediated transcriptions of various genes encoding the above-mentioned antioxidant enzymes [21]. In addition, PCs can also suppress oxidative stress by reducing inflammatory responses via interfering with nuclear factor kappa B (NFkB) and mitogen-activated protein kinase (MAPK)-controlled inflammatory signalling cascades [22] whose activation leads to the innate magnification of regulatory immune responses. As a result, pro-inflammatory cytokines, including interleukin (IL)-1b, IL-6, IL-8, tumor necrosis factor (TNF)-α, and interferon (IFN)-γ, are released into the circulation system, which, if not properly regulated, can trigger irreversible systemic inflammation and disrupted immune homeostasis. 

PCs obtained both from grape skin and seeds are also able to modulate the function of several matrix metalloproteinases (MMPs), also called gelatinase, belonging to the family of zinc-dependent enzymes with endopeptidase activity which are involved in a wide range of physiological and pathological events associated with the turnover of the extracellular matrix. In the case of ruminants, matrix metalloproteinase (MMP)-9 (or gelatinase B) has been characterized for its role in remodeling and tissue regeneration during the dry period, allowing for the regeneration of the mammary gland and guaranteeing an optimal milk production in the subsequent lactation [23]. However, the presence of this enzyme in milk of cattle, goats, and ewes, together with MMP-2 (or gelatinase A), have been associated with variations in the number of milk somatic cells as a consequence of the onset of mastitis events of various natures and extent also in the absence of a clear clinical symptomatology [24,25,26]. The presence and the activity of these enzymes in milk represents an indicator of the animal’s physiology. Furthermore, in a recent study on ewes’ milk, the MMP-9 caseinolytic potential has been evidenced by bioinformatics tools [25]. A high presence or activity of this enzyme in the milk could therefore cause an excessive casein proteolysis, affecting the technological properties of milk, the dairy yield, and the nutritional and sensory quality of dairy products. 

Our hypothesis was that supplementing the diet of lactating ewes with GP might enrich the milk of PCs able to improve the milk nutraceutical characteristics, and in addition, modify antioxidant and inflammatory milk indicators. 

## 2. Materials and Methods

The experimental design was set up taking into account the Directive 2010/63/EU of the European Parliament [27] and Directive 86/609/EEC [28], which deals with the protection of animals used for scientific purposes. The trial was performed in a dairy sheep farm in which no alternative breeding practices have been introduced to those already adopted by the farmer. For this reason, there are no ethical issues to be addressed.

### 2.1. Animals and Milk Sampling

This study is part of a project studying the effects of a diet containing 10% GP on the quality of milk and derived dairy products. Details on the experimental design have been reported in a previous study [29]. Briefly, the study was performed in a farm located in Central Italy (Roseto degli Abruzzi (TE), Italy) and involved 46 crossbreed dairy ewes homogeneous for milk yield and body weight. Ewes were then randomly assigned to two groups of 23 animals each: a control group (Ctrl) that received a standard diet and an experimental group (GP+) whose diet was been formulated with 10% GP on a dry matter (DM) basis. Each animal specifically received alfalfa hay ad libitum in addition to 1 kg per day of a custom formulated concentrate (CFC) that, in the case of GP+, was characterized by dietary supplementation. The CFC was administered to ewes in two daily rations of 500 g each, concurrently with the two milkings in the morning (8:00) and in the evening (18:00). The trial lasted 70 days, including an initial adaptation period of 10 days. Samples of CFC were taken and analyzed for chemical composition, total phenolic content, and antioxidant activity as previously reported by Bennato et al. [29], and for phenolic profile characterization. At the end of the trial, milk samples from the Ctrl group (*n* = 10) and GP+ group (*n* = 10) were randomly selected, aliquoted, and immediately frozen at −20 °C before carrying out analysis for the characterization of the phenolic profile, the determination of antioxidant and inflammatory status, gelatinase activity, and MMPs activity and expression.

### 2.2. Phenolic Profile Characterization of Custom Formulated Concentrate

The standards of phenolic compounds used in this research were: gallic acid, OH-tyrosol, protocatechuic acid, (-)-epigallocatechin, 3-hydroxybenzoic acid, tyrosol, chlorogenic acid, epicatechin, caffeic acid, vanillic acid, catechin, (-)-epigallocatechin gallate, syringic acid, orientin (luteolin-8-glucoside), rutin, p-coumaric acid, hyperoside (querc-3-d-galactoside), isoquercetin (querc-3-b-d-glucoside), ferulic acid, hesperidin, rosmarinic acid, oleuropein, o-coumaric acid, sinapic acid, myricetin, luteolin, quercetin, trans-cinnamic acid, naringenin, isoxanthumol, apigenin, diosmetin (luteolin-4-methyl ether), kaempferol, and xanthohumol; they were purchased from Sigma-Aldrich (Saint Louis, MO, USA). The working standard mixtures were prepared by appropriate dilution in methanol and stored at −20 °C. Ultrapure water, acetic acid, formic acid, methanol, and acetonitrile were ultra-pure grade and were purchased from VWR (Radnor, PA, USA).

For the liquid chromatography mass-spectrometry (LC-MS/MS) analysis, 100 mg of CFC were homogenized in 1 mL methanol:water (80:20, *v*/*v*) with formic acid (0.5%, *v/v*). The samples were extracted with Precellys^®^ Evolution homogenizer (Bertin Technologies SAS, Montigny-le-Bretonneux, France at 6500 rpm with 10 s pause (3 times) for 30 s and centrifuged at 10,000 rpm for 10 min at 4 °C. The supernatant was withdrawn, and the pellet was extracted again under the same conditions. The extracts, for each sample, were combined and purified by SPE for clean-up step. The elutions were diluted in a 1:100 ratio and injected into LC-MS/MS for analysis.

For targeted analysis of CFC, a Nexera XR HPLC system (Shimadzu, Tokyo, Japan) coupled to a 4500 Qtrap mass spectrometer (Sciex, Toronto, ON, Canada) equipped with a heated ESI source (source V) was used, following Oliva et al. [30]. An Excel 2 C18-PFP (10 cm × 2.1 mm ID) column from Advanced Chromatography Technologies (Aberdeen, UK) with 2 μm particles and safety protection was used for the separation of the analytes. The mobile phases were water with acetic acid (1%, *v/v*) (A) and acetonitrile (B). The flow rate was set to 0.400 mL/min and the injection volume was set to 6 µL. All analytes were detected in negative ionization with a capillary voltage of −4500 V, nebulizer gas (air) at 40 psi, and turbogas (nitrogen) at 40 psi and 500 °C. Data collection and processing were performed with Analyst 1.7.2 software and quantification with Multiquant 3.0 (Sciex) software.

### 2.3. Milk Phenolic Profile Characterization

For the characterization of the phenolic profile, milk samples were previously centrifugated at 4000 rpm × 10 min to eliminate the fat and further protein precipitation. The defatted samples were extracted following the procedure of Branciari et al. [31] with some modifications. Briefly, 10 mL of each sample was collected in a falcon and 150 µL of citric acid (1.5 M) was added to further casein precipitation. Then, 1 mL of each sample was collected in an eppendorf with 4 mL of methanol; the samples were then centrifuged at 10,000 rpm for 10 min at 4 °C. The supernatant was collected and re-extracted the pellet with 2 mL of a methanol:water (80:20, *v/v*) mixture containing 37.5 µL of ascorbic acid. The extract was then vortexed and centrifuged under the same conditions. The supernatants obtained were combined with the previous ones, and 5 mL of each were collected and brought to pH 1 with hydrochloric acid (25%, *v/v*). Subsequently, the extracts were centrifuged again, 1 mL of each was withdrawn, and 7 mL of water acidified to pH 7 was added. The samples were then purified using the clean-up method, as described in the previous article by Oliva et al. [30]; the extracts were then loaded onto SPE and 1 mL of a phosphate buffer mixture (50 mM) at pH 3: methanol (90:10, *v/v*) was added; then the resulting solution was loaded onto a Stata XL cartridge (330 mg, 1 mL) from Phenomenex (Torrance, CA, USA). The cartridge was then eluted with 1 mL of methanol. The samples were then dried with the SpeedVac (SPD120, ThermoFisher Scientific, Greenville, NC, USA), resumed with 100 µL of methanol, and injected into LC-MS/MS for the identification and quantification of phenolic compounds as previously reported in Section 2.2.

### 2.4. Enzyme-Linked Immunosorbent Assay of Interleukin-1, Glutathione Peroxidase, Catalase and Tumor Necrosis Factor in Milk

For the determination of antioxidant and inflammatory status, defatted milk samples were analyzed with commercial kits (MyBioSource, Inc., San Diego, CA, USA) in order to determine the amount of IL-1 (MBS034397), TNFα (MBS2019710), GPx (MBS841725), and CAT (MBS034397) activities. The levels of IL-1 and TNFα were determined with an ELISA kit based on antibody–antigen interactions and an HRP colorimetric detection system to detect the antigen targets in samples. Both the levels of IL-1 and TNFα were measured at 450 nm, and both expressed in pg/mL. GPx activity was determined indirectly by measuring the formation rate of oxidized glutathione (GSSG). Glutathione peroxidase activity was measured spectrophotometrically at 412 nm as decreased absorbance and GPx activities were expressed as nmol/min/mL. Catalase activity was evaluated using a kit that utilizes the peroxidase function of catalase for measuring catalase activity. In the presence of an appropriate concentration of H_2_O_2_, catalase reacts with methanol to produce formaldehyde, which can react with chromogens. The absorbance of the product was measured at 540 nm photometrically. The results are expressed as nmol/min/mL. The measurements were performed using an ELISA microplate reader (EnSpire 2300 multireader; PerkinElmer, Waltham, MA, USA).

### 2.5. Milk Gelatinase Activity

Total gelatinolytic activity of Type IV collagenases (MMP-2/MMP-9) of defatted milk samples was obtained through a fluorometric method based on the use of a specific synthetic self-quenched substrate, MOCAc-Pro-Leu-Gly-(2-mercapto-4-methylpentanoyl)-Leu-Gly-OEt (Merck Life Science S.r.l., Milan, Italy), as previously reported by Ianni et al. [25]. The substrate cleavage was monitored at 324 nm for 10 min at a constant temperature of 38 °C through a LS-50B luminescence spectrophotometer (PerkinElmer, Milan, Italy) equipped with thermostatically controlled cells. The mix of reaction was composed by a 5 µL sample and 5 µM substrate in 50 mM Tris-HCl pH 8.0, containing 5 mM CaCl_2_, 200 mM NaCl, and 0.03% Brij L23 (Sigma-Aldrich, Milan, Italy). Data were reported as units of fluorescence (UF)/min/mg total protein.

### 2.6. Milk Zymographic Analysis of Matrix Metalloproteinases

The zymographic evaluation of gelatinases (MMP-2 and MMP-9) were performed on defatted milk samples, as previously reported by Ianni et al. [25]. The proteins were quantified using the Bradford method [32] and Bovine Serum Albumine (BSA) as the standard. Volumes of each sample corresponding to 15 µg of total proteins were diluted in a non-reducing sample buffer (62.5 mM Tris-HCl pH 6.8, 2% SDS, 10% glycerol, and bromophenol blue) without heating and resolved by 8% SDS-PAGE containing 0.15 mg mL^−1^ type B gelatin (Sigma Aldrich, Milan, Italy). Molecular weight markers in the range of 250–10 kDa (Biorad, Milan, Italy) were run on SDS-PAGE in parallel with samples for molecular weight estimation. The gels were then incubated for 45 min in a renaturation buffer (50 mM Tris-HCl pH 8.0, containing 2.5% Triton X-100) to remove SDS. Subsequently, a 24 h incubation in the developing buffer (50 mM Tris-HCl pH 8.0, containing 5 mM CaCl_2_, 200 mM NaCl and 0.02% Brij 35) was performed to allow enzyme renaturation. The gels were then stained in a 0.1% solution of coomassie blue R250 in 40% (*v*/*v*) methanol and 10% (*v*/*v*) acetic acid. In preliminary evaluations, the supernatant of HT1080 fibrosarcoma cells was used as a reference standard for both MMP-2 and MMP-9. Furthermore, additional tests were performed in order to verify the metalloprotease nature of the observed activity. With this purpose, the zymographic gels were incubated also in a developing buffer in which there was the addition of a chelating agent (EDTA 100 mM); no hydrolysis of the substrate was found in this condition. The intensity of the corresponding bands was determined using ImageLab 6.0.1 software.

### 2.7. Metalloproteinase-9 Protein Expression

MMP-9 protein expression was evaluated on samples of skimmed milk containing 20 µg total protein, determined by Bradford methods [32]. Milk samples were mixed with a reducing sample buffer (62.5 mM Tris-HCl pH 6.8, 2% SDS, 10% glycerol, 5% β-mercaptoethanol and bromophenol blue), boiled for 5 min, and subjected to 10% SDS-PAGE. As reported for zymographic analysis, molecular weight markers in the range of 250–10 kDa were run on SDS-PAGE in parallel with samples for molecular weight estimation. The proteins, separated by SDS-PAGE, were then transferred for 1 h at a constant voltage of 100 V onto polyvinylidene difluoride (PVDF) membranes. Subsequently, the membranes were blocked using EveryBlot Blocking Buffer (Biorad, Milan, Italy) for 5 min with agitation at room temperature, washed in TBS-T (10 mM Tris-HCl pH 8; 150 mM NaCl; 0.1% Tween 20), and incubated overnight at 4 °C with the primary antibodies for human MMP-9 (Santa Cruz Biotechnology, Santa Cruz, CA, USA), after verification of cross reactivity with sheep. The primary antibody was diluted 1:500 in EveryBlot Blocking Buffer. Membranes were then washed in TBS-T and incubated for 1 h with secondary HRP-conjugated antibody diluted 1:1000 in EveryBlot Blocking Buffer. The immunoreactive bands were detected by inducing a chemiluminescence reaction through the ECL chemiluminescent reagent (GE Healthcare, Little Chalfont, England). Quantitative analysis of immunoreactive spots was performed as previously described for zymography.

### 2.8. Molecular Docking

The possible interaction between MMP-9 and luteolin was evaluated through an in silico approach using SwissDock, a web service developed by the Swiss Institute of Bioinformatics (SIB) to predict the most favorable binding modes that may occur between a target protein and a ligand. This web tool bases its own assessments on the docking software EADock DSS [33] and the CHARMM force field method for calculation [34]. As a protein model, the crystal structure of human MMP-9 (chain A) complexed with a reverse hydroxamate inhibitor (Protein Data Bank (PDB) code: 1GKC) [35] was used, since no crystallized structures are available for ruminants. The docking clusters related to the most favorable interactions between MMP-9 and luteolin were visualized and analyzed using the software UCSF Chimera 1.11.2 [36].

### 2.9. Statistical Analyses

All experiments were performed at least in triplicate, and all 10 milk samples of each group were analyzed. The JMP Pro 14 program (SAS Institute, Cary, NC, USA) was used to process data. The Student’s *t*-test was applied in order to identify significant differences between the two groups. Statistical significance was attributed to *p* lower than 0.05. Data were reported as least square means ± standard error of the mean (SEM). 

## 3. Results

### 3.1. Diet Phenolic Profile

**Figure 1:**
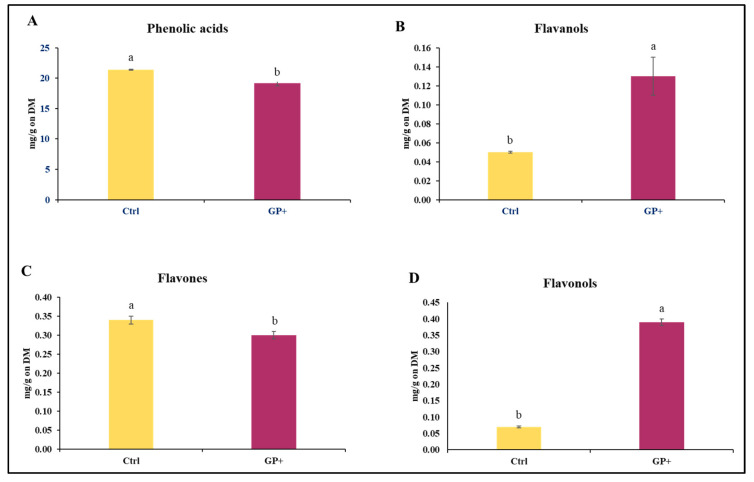
Main classes of phenolic compounds: (**A**) phenolic acids, (**B**) flavanols, (**C**), flavones, and (**D**) flavonols, identified in the custom formulated concentrate administered to the control group (Ctrl) and experimental group (GP+). ^a,b^ Means with different uppercase superscript letters are significantly different (*p* < 0.05). All data are reported as least square means ± SEM and expressed on a Dry Matter (DM) basis.

With regard to the CFC administered to the ewes, a lower content of phenolic acids (Figure 1A) and flavones (Figure 1C) and higher levels of flavanols (Figure 1B) and flavonols (Figure 1D) were found in the GP+ diet. 

Specifically, 23 compounds were identified, and significant differences were found between the two CFCs (Table 1). The GP+ CFC had a higher quantity of phenolic acids such as gallic acid (*p* < 0.001), syringic acid (*p* < 0.01), ellagic acid (*p* < 0.01), protocatechuic acid (*p* < 0.01), chlorogenic acid (*p* = 0.01), and 4-hydroxybenzoic acid (*p* = 0.03), of flavanols such as epicatechin (*p* = 0.02), flavonols such as quercetin (*p* = 0.01), quercetin-hexoside (*p* < 0.01), and myricetin (*p* < 0.001), flavones as naringenin (*p* = 0.03) and orientin (*p* < 0.001), and a lower quantity of flavones such as diosmetin (*p* = 0.01).

### 3.2. Milk Phenolic Characterization

**Figure 2:**
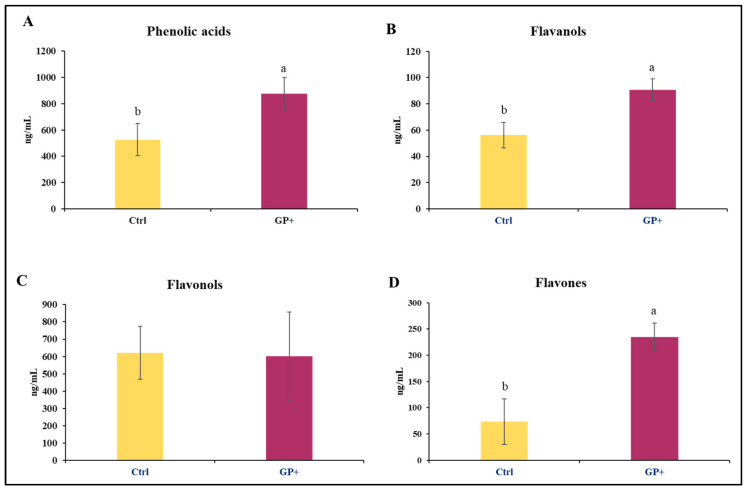
Main classes of phenolic compounds: (**A**) phenolic acids, (**B**) flavanols, (**C**) flavonols, and (**D**) flavones, identified in raw milk samples obtained from the control group (Ctrl) and experimental group (GP+). ^a,b^ Means with different uppercase superscript letters are significantly different (*p* < 0.05). All data are reported as least square means ± SEM; (*n* = 10 each group).

The analyses performed on raw milk samples at the end of the trial allowed for the identification of 22 compounds belonging to phenolic acids, flavanols, flavonols, and flavones. Higher levels of phenolic acids (Figure 2A), flavanols (Figure 2B), and flavones (Figure 2D) were found in GP+ milk samples; conversely, the levels of flavonols (Figure 2C) were almost similar between the two groups.

Most of the identified compounds were presented in the diet. However, as reported in Table 2, in addition to the compounds found in the diets, in milk samples were also identified epigallocatechin, epigallocatechin gallate, caftaric acid, rosmarinic acid, trans-cinnamic acid, and OH-tyrosol. Therefore, the diet with GP affected the content of phenolic compounds in the milk. In particular, in GP+ milk samples, higher levels of luteolin (*p* = 0.01), kaempferol (*p* = 0.03), epigallocatechin gallate (*p* = 0.02), rosmarinic acid (*p* = 0.03), and OH-tyrosol (*p* = 0.02) were found. 

### 3.3. Milk Antioxidant and Inflammatory Status

**Figure 3:**
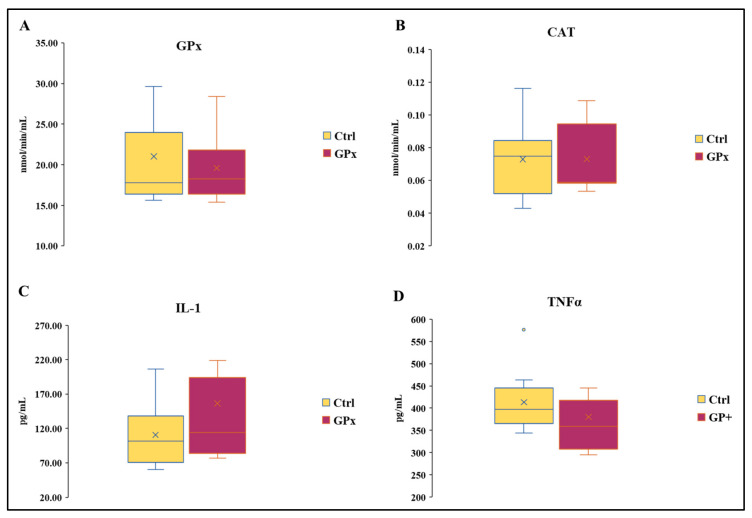
Box plot showing (**A**) catalase (CAT) and (**B**) glutathione peroxidase (GPx) activity and IL-1 (**C**) and TNFα (**D**) levels in the raw milk of ewes fed a standard diet (Ctrl) and grape pomace diet (GP+). Differences between Ctrl and GP+ were not significant (*p* > 0.05); (*n* = 10 each group).

There was no effect of the diet on the activity of GPx and CAT (Figure 3A,B). The activity of GPx in Ctrl milk ranged from 15.61 to 38.30 nmol/min/mL and from 15.37 to 28.42 nmol/min/mL in GP+. Likewise, the activity of catalase was similar between the two groups and ranged from 0.05 to 0.12 nmol/min/mL in Ctrl and from 0.05 to 0.11 in GP+. No statistically significant differences were found in IL-1 concentration between the two groups, whose levels ranged from 60 to 206.15 pg/mL and from 76.92 to 426.92 pg/mL, respectively, in Ctrl and GP+ milk samples (Figure 3C). The levels of TNF1 ranged from 3.44 to 5.27 ng/mL in Ctrl milk samples, while they ranged from 2.95 to 6.13 ng/mL in GP+ milk samples, and no statistically significant differences were found between the two groups (Figure 3D). 

### 3.4. Evaluation of the Overall Gelatinolytic Activity in Milk

As reported in Table 3, the values related to the enzymatic activity did not show significant differences between the two groups, even if the GP+ value was found to be slightly lower compared to Ctrl, and a *p* value not far from statistical significance should be underlined (*p* = 0.052). 

### 3.5. Milk Metalloprotease Activity and Expression

**Figure 4:**
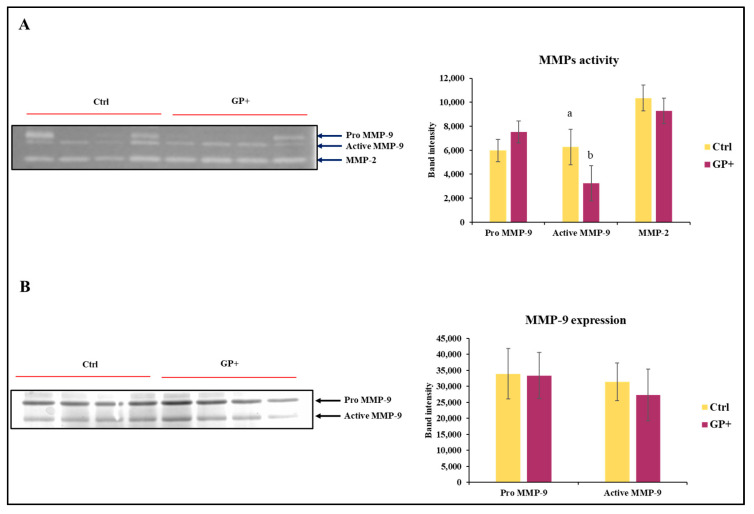
Representative zymography (**A**) of the inactive form of the enzyme MMP-9 (Pro MMP9), the active form of MMP-9 (Active MMP9) and MMP-2 activity of raw milk samples obtained from ewes fed a standard diet (Ctrl) and grape pomace diet (GP+). Representative Western blot (**B**) evaluation of the inactive form of the enzyme MMP-9 (Pro MMP-9) and the active form of MMP-9 (Active MMP-9). The histograms respectively report the magnitude intensity of Pro MMP-9, Active MMP-9, and MMP-2 activity (**A**) and the amount of Pro MMP-9 and Active MMP-9 (**B**) in the milk. The values were obtained from the densitometric analysis of the corresponding bands and were reported as band intensity. ^a,b^ Means with different uppercase superscript letters are significantly different (*p* < 0.05). All data are reported as least square means ± SEM; (*n* = 10 each group).

The zymographic analysis was performed to discriminate the enzymatic activities attributable to MMP-9 and MMP-2. With regard to MMP-9, this approach allows for the identification of not only the active form of the enzyme (86 kDa), but also the pro-enzymatic form (Pro MMP-9; 92 kDa), characterized by a mild hydrolytic activity (Figure 4A). In regard to the activity of Pro MMP-9 and MMP-2, no statistically significant differences were found between the two groups. Conversely, MMP-9 was less active in GP+ milk samples compared to the Ctrl. After evaluating the activity of MMP-9, it was considered appropriate to analyze the presence of the enzyme with an immunorecognition method (Figure 4B). This approach did not show differences between the two groups in the expression of MMP-9.

### 3.6. In Silico Evaluations of the Interaction between Luteolin and MMP-9

**Figure 5:**
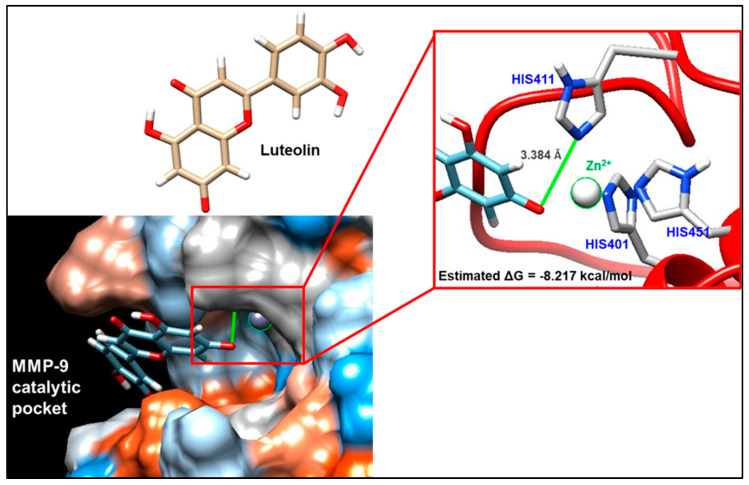
Molecular docking evaluation of the interaction between matrix metalloproteinase 9 (MMP-9) and luteolin. The interaction was analyzed by exploiting the ViewDock tool of the UCSF Chimera software. View of the hydrophobic surface of the enzyme through which it is possible to visualize the most plausible mode of interaction, for which a free energy value (ΔG) equal to −8.217 kcal/mol was highlighted. The interaction foresees that luteolin approaches the catalytic pocket of the enzyme, placing some hydroxyl residues of its structure in proximity to the catalytic zinc that is coordinated by histidines 401, 411, and 451.

In order to predict the most probable binding conformations between luteolin and MMP-9, a preliminary in silico study was performed. The most favorable docking is associated with a ΔG value equal to −8.217 kcal/mol; in this condition (Figure 5), luteolin is able to approach the catalytic pocket of the enzyme, placing some hydroxyl residues of its structure in proximity to the catalytic zinc that is coordinated by histidines 401, 411, and 451. In addition to this, the distance between luteolin and the catalytic zinc ion is 3.384 Å, suggesting the plausible existence of a non-covalent interaction.

## 4. Discussion

The feeding strategy can affect the presence of PCs in the milk [37,38]. However, the degree to which PCs can be transferred from feed to animal products is not well known. The physicochemical properties of PCs, such as molecular weight, glycosylation, and esterification, influence their intestinal absorption [39,40], and several studies on the bioavailability of polyphenols in ruminants indicate that the digestive system of ruminants absorbs only small amounts of these compounds [17].

In our experiment, the characterization of the phenolic profile through an ultra-high-performance liquid chromatography system showed a different profile both between the diet administered to ewes and in milk samples. In particular, the enrichment with GP resulted in the presence of significantly higher levels of flavanols and flavonols and lower quantities of phenolic acids and flavones compared to the Ctrl diet. Many dietary PCs were even present in the milk, suggesting the plausibility of a direct transfer. Conversely, some compounds present in the milk were not detected in the diets and vice versa; this could be a consequence of different mechanisms of absorption and/or the occurrence of metabolic transformations. In fact, the reciprocal interaction between PCs and rumen microbiota can affect their bioaccessibility. The different CFC phenolic profile could alter the levels of several parameters related to rumen fermentation (e.g., pH and concentration of fermentation products in rumen juice) and modulate the composition and the activity of bacterial community in the rumen, and in addition, the bacteria diversity could lead to a different biotransformation of the polyphenols themselves [41].

Conversely to what was observed in the diet, in GP+ milk samples, higher levels of phenolic acids, flavanols, and flavones were found. In general, low-weight polyphenols such as phenolic acids or several flavonoid aglycones can be absorbed by passive diffusion [42]. In fact, a lot of phenolic acids found in the CFC were also detected in the milk samples of both groups even if among phenolic acids, rosmarinic and ferulic were present at higher concentration in GP+ milk compared to Ctrl milk samples. The absence of rosmarinic acid in the diet and, on the contrary, its presence in the milk could be a consequence of metabolic transformation of other phenolic acids.

In plant species, it has been shown that both rosmarinic and ferulic acids are produced starting with caffeic acid [43]. Rosmarinic acid is an ester of caffeic acid and ferulic acid is produced starting with L-tyrosine and caffeic acid 3-O-methyltransferase, which mediates the methylation of caffeic acid to ferulic acid. However, their biosynthesis in ruminant is not known. 

Among flavonols, higher levels of epigallocatechin gallate, kaempferol, and luteolin were found in GP+ samples. The fact that these compounds were present at higher levels in GP+ samples represents an important finding in the potential benefits for consumers’ health. Epigallocatechins, such as other catechins, have many reported biological activities, such as antioxidative, anticarcinogenic, and antitumor properties [44,45,46]. Luteolin plays a key role in biological activities, including antioxidant activity, cardiovascular protection, eye health, and skin health [47,48]. Kaempferol is known to be one of the most active and important natural anti-inflammatory compounds [49,50].

Taking into account the presence of these compounds in milk and their bioactive functions, it was deemed useful to evaluate in milk the antioxidant and inflammatory status. First of all, the activity of GPx and CAT and the levels of IL-1 and TNFα were investigated. The results did not show significant differences between the milk samples of the two groups, testifying to the fact that diet did not influence the expression and/or the activity of these proteins. This finding may suggest that the ewes belonging to both groups were effectively in good health and positive energy balance, and changes in antioxidant activity and inflammatory levels could emerge only when animals were under oxidative stress. 

The aspect regarding the inflammatory condition has been further evaluated by monitoring the presence and activity of proteases physiologically involved in the remodeling processes of the extracellular matrix, which tend to be overexpressed in the presence of tissue lesions. In particular, reference was made to metalloproteinases 2 and 9, belonging to the gelatinase family. The spectrofluorimetric analysis of the overall enzymatic activity of the gelatinases showed a slightly lower value, not far from statistical significance (*p* = 0.052), in GP+ milk compared to Ctrl milk, suggesting a possible different proteolytic activity of MMPs. Only the zymographic analysis, performed to discriminate between the enzymatic activities deriving from the two major gelatinases, MMP-2 and MMP-9, allowed for highlighting lower levels of MMP-9 activity in GP+ samples but not significant differences in MMP-2 activity. Both MMPs are involved in physiological and pathological events; however, MMP-2 when compared with MMP-9 is less subject to changes also in the presence of inflammatory stimuli [51]. To better understand if the lower activity was associated with a lower protein expression, western blotting analysis on MMP-9 was performed. However, no significant differences between the two groups were found, supposing the hypothesis of a direct inhibition mechanism correlated to the interaction between milk compounds and the enzyme. Many studies on human cell models have shown the ability of PCs to reduce the enzymatic activity of MMPs. Green tea polyphenol epigallocatechin-3-*O*-gallate (EGCG) inhibits tumor invasion by directly inhibiting the MMP-2/-9 activity [52]. In human fibrosarcoma cells, a specific mixture of polyphenols inhibited both MMP-2 and -9 secretion in a dose-dependent manner, with total inhibition at 50 μg/mL [53]. In subsequent treatment with luteolin, a decrease in the expression of MMP-2 and MMP-9 in azoxymethane (AOM)-induced colon carcinogenesis in Balb/C mice was found. The ability of flavonoids to inhibit the MMP-9 activity interacting with the MMP-9 catalytic domain has been also reported for quercetin [54]. Nevertheless, even though the relationship between flavonoid chemical structure and the inhibitory property on MMP activity has been established, the molecular mechanisms of this inhibition are still unknown [55]. In the present work, by using molecular modelling techniques, such as molecular docking and dynamics simulations, it has been possible to identify the putative interaction sites involved in the direct inhibition of active MMPs by luteolin, one of the most represented PCs found in GP+ milk. Our data clearly indicate the luteolin’s ability to interact with MMP-9. In particular, luteolin showed to be able to approach the catalytic pocket of the enzyme, placing a hydroxyl residue of its structure in proximity to the catalytic zinc that is coordinated by histidines 401, 411, and 451. Specifically, it seems that luteolin may generate a non-covalent interaction directly with the catalytic zinc, which would be at the center of a tetrahedral structure, which would make it difficult for it to interact with the substrate. This condition is part of a competitive inhibition model, and, in this regard, it should be underlined what has been previously reported by Ende and Gebhardt [56] who collected evidence to support a non-competitive inhibition model. This observation would therefore presuppose further and more in-depth evaluations.

## 5. Conclusions

In conclusion, our data constitute evidence that the use of agro-industrial byproducts rich in polyphenols in the feeding and nutrition of dairy ewes can be considered as a useful strategy to improve the content of bioactive compounds in the milk. In addition, the presence of these compounds can modulate the activity of MMP-9, an important enzyme involved in physiological and pathological events and whose excessive presence in the milk can affect the technological properties of milk, the dairy yield, and the nutritional and sensory quality of dairy products. 

## Figures and Tables

**Table 1:** Phenolic compounds identified in the custom formulated concentrate administered to the control group (Ctrl) and experimental group (GP+).

	Ctrl (*n* = 3)	GP+ (*n* =3)	*p*
Phenolic acids ^1^, mg/g			
caffeic acid	0.11 ± 0.01	0.07 ± 0.02	0.11
chlorogenic acid	14.92 ± 0.03	12.02 ± 0.41	0.01
ellagic acid	0.01 ± 0.01	0.02 ± 0.00	<0.01
ferulic acid	0.05 ± 0.00	0.05 ± 0.01	0.61
gallic acid	<LOQ	0.05 ± 0.00	<0.001
p-coumaric acid	0.02 ± 0.00	0.03 ± 0.00	0.10
protocatechuic acid	4.23 ± 0.08	5.54 ± 0.01	<0.01
sinapic acid	0.03 ± 0.00	0.04 ± 0.00	0.12
siringic acid	0.02 ± 0.00	0.08 ± 0.00	<0.01
vanillic acid	0.06 ± 0.00	0.08 ± 0.01	0.06
4-hydroxybenzoic acid	1.92 ± 0.00	1.20 ± 0.17	0.03
Flavanols ^1^, mg/g			
catechin	0.05 ± 0.00	0.06 ± 0.00	0.13
epicatechin	<0.01 ± 0.00	0.07 ± 0.01	0.02
Flavonols ^1^, mg/g			
kaempferol	<0.01 ± 0.00	<0.01 ± 0.00	0.15
myricetin	<0.01 ± 0.00	<0.01 ± 0.00	<0.001
quercetin	0.04 ± 0.00	0.23 ± 0.02	0.01
quercetin-hexoside	0.02 ± 0.00	0.15 ± 0.01	<0.01
rutin	0.01 ± 0.00	0.01 ± 0.00	0.25
Flavones ^1^, mg/g			
apigenin	<0.01 ± 0.00	<0.01 ± 0.00	0.49
diosmetin	0.31 ± 0.01	0.17 ± 0.01	0.01
luteolin	<0.01 ± 0.00	<0.01 ± 0.00	0.06
naringenin	<0.01 ± 0.00	<0.01 ± 0.00	0.03
orientin	0.01 ± 0.00	0.12 ± 0.00	<0.001

^1^ All data are reported as least square means ± SEM and expressed on a Dry Matter (DM) basis.

**Table 2:** Phenolic compounds identified in raw milk samples obtained from the control group (Ctrl) and experimental group (GP+).

	Ctrl (*n* = 10)	GP+ (*n* = 10)	SEM	*p*
Phenolic acids, ng/mL				
caftaric acid	37.28	65.29	25.70	0.25
caffeic acid	129.05	148.71	52.97	0.66
chlorogenic acid	4.78	7.44	2.34	0.19
ellagic acid	76.71	124.78	34.86	0.19
ferulic acid	11.20	17.37	3.37	0.05
gallic acid	64.13	87.67	36.35	0.45
p-coumaric	17.89	30.01	10.61	0.16
protocatecuic acid	9.45	12.59	2.26	0.11
rosmarinic acid	101.83	280.21	78.18	0.03
siringic acid	2.88	4.86	2.39	0.30
trans-cinnamic acid	1.15	2.11	0.50	0.08
vanillic acid	60.37	78.43	25.88	0.42
4-hydroxybenzoic acid	9.87	17.01	4.20	0.11
Flavanols, ng/mL				
catechin	9.84	27.56	10.59	0.11
epicatechin	0.31	0.66	0.36	0.34
epigallocatechin	43.39	53.36	5.82	0.07
epigallocatechin gallate	2.68	9.20	2.55	0.02
Flavonols, ng/mL				
kaempferol	28.08	60.37	13.39	0.03
quercetin	593.50	541.80	295.56	0.87
Flavones, ng/mL				
luteolin	39.39	123.19	31.10	0.01
naringenin	33.95	111.07	44.64	0.09

All data are reported as least square means ± SEM.

**Table 3:** Gelatinolytic activity in the raw milk of ewes fed a standard diet (Ctrl) and grape pomace diet (GP+).

	Ctrl (*n* = 10)	GP+ (*n* = 10)	SEM	*p*
Gelatinase activity, UF/min/mg	1.13	0.82	0.14	0.052

Data are reported as least square means ± SEM.

## Data Availability

The data of this study are available on request from the corresponding author, [G.M.].
